# Platelet count predicts mortality in patients with sepsis: A retrospective observational study

**DOI:** 10.1097/MD.0000000000035335

**Published:** 2023-09-22

**Authors:** Yusi Hua, Ruoran Wang, Jie Yang, Xiaofeng Ou

**Affiliations:** a Department of Anesthesiology, West China Hospital, Sichuan University, Chengdu, Sichuan, China; b Department of Neurosurgery, West China Hospital, Sichuan University, Chengdu, China; c Department of Critical Care Medicine, West China Hospital, Sichuan University, Chengdu China.

**Keywords:** mortality, platelet count, sepsis, thrombocytopenia

## Abstract

Platelet count is a key component of sepsis severity score. However, the predictive value of the platelet count at admission for mortality in sepsis remains unclear. We designed a retrospective observational study of patients with sepsis admitted to our hospital from January 2017 to September 2021 to explore the predictive value of platelet count at admission for mortality. A total of 290 patients with sepsis were included in this study. Multivariate logistic regression analysis was used to evaluate the risk factors for mortality and construct a predictive model with statistically significant factors. Compared with survivors, nonsurvivors tended to be much older and had significantly higher acute physiology and chronic health evaluation II and sequential organ failure assessment scores (*P* < .001). The platelet count was significantly lower in the nonsurvivor group than in the survivor group (*P* < .001). Multivariate logistic regression analysis indicated that age (*P* = .003), platelet count (*P* < .001) and lactate level (*P* = .018) were independent risk factors for mortality in patients with sepsis. Finally, the area under the receiver operating characteristic curve of platelet count predicting mortality in sepsis was 0.763 (95% confidence interval, 0.709–0.817, *P* < .001), with a sensitivity of 55.6% and a specificity of 91.8%. In our study, platelet count at admission as a single biomarker showed good predictability for mortality in patients with sepsis.

## 1. Introduction

Sepsis is defined as a life-threatening organ dysfunction caused by dysregulated host response to infection.^[1]^ Despite significant advances have been made in sepsis management, it remains a major health care challenge with high global incidence and deaths each year.^[2,3]^ Early identification and prediction of sepsis progression play an important role in the clinical course and outcomes.^[4–6]^ Many efforts have been made to discover predictable markers of the severity and mortality of patients with sepsis in the early stages.^[7,8]^

During sepsis, thrombocytopenia (TP) is a common complication in the intensive care unit (ICU), as the platelet count drops in sepsis due to its consumption and dysfunction in inflammation and thrombosis.^[9,10]^ Persistent TP is an independent risk factor for mortality and reflects disease severity of sepsis.^[11]^ Therefore, several markers of platelets, especially many combined biomarkers, have been investigated to predict mortality and disease severity in sepsis; for example, combining the mean platelet volume to platelet count ratio is a promising predictor of early mortality in severe sepsis.^[12]^ In addition, a scoring system using red blood cell distribution width, delta neutrophil index, and mean platelet volume to platelet count is a better predict markers for mortality in patients with sepsis.^[13]^ Although some combined markers have been found to predict mortality in critical patients, they are not widely available in clinical practice, perhaps because they are tedious and not as convenient as individual biomarkers. Therefore, it is necessary to identify a reliable and widely available biomarker. We designed this study to verify the hypothesis that the platelet count at ICU admission could predict mortality in patients with sepsis.

## 2. Materials and Methods

### 2.1. Setting and study population

This study was approved by the Ethics Committee of the West China Hospital (No.2021-1713). This retrospective observational study was conducted from January 2017 to September 2021 at West China Hospital, a tertiary teaching hospital in Southwest China. We identified and enrolled patients diagnosed according to the latest third international definition of sepsis (Sepsis-3).^[1]^ The following criteria were used to exclude patients: age < 18 years, acute bleeding, missing data on hospital mortality and platelet count at admission, hematological malignancy and administration of any affecting drugs (such as granulocyte colony-stimulating factors or corticosteroids). In addition, we did not include patients with a length of ICU stay of < 24 hours and were not followed up.

**Figure 1.**
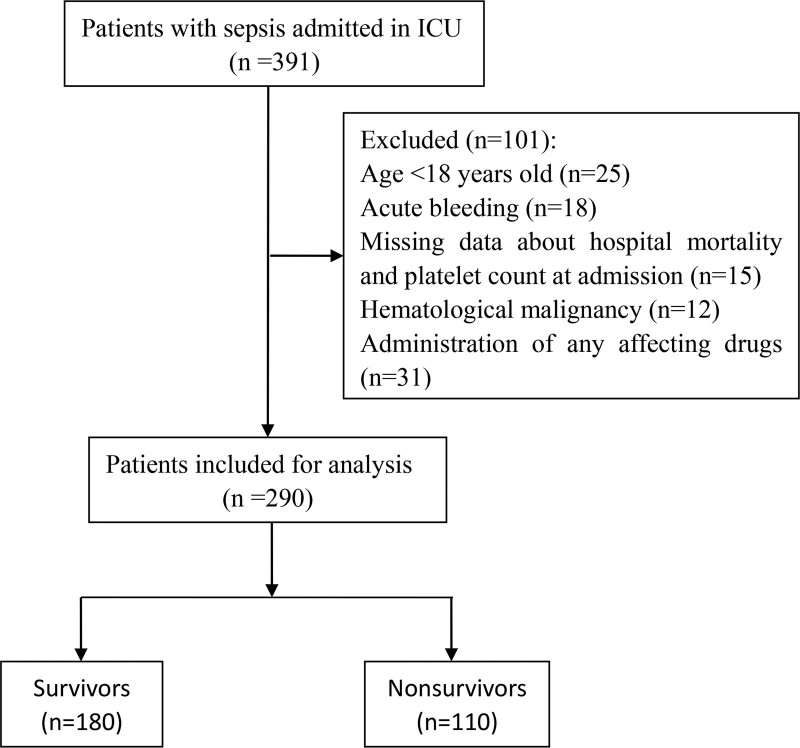
Flowchart diagram of patient enrollment. ICU = intensive care unit.

The flow of the inclusion process is illustrated in Figure 1. Of the 391 patients recruited for this study, 127 were excluded based on the exclusion criteria. Finally, 290 patients were included in this study.

### 2.2. Data collected

All clinical and laboratory data of each subject were extracted from the electronic medical record system of West China Hospital, and follow-up information was recorded by a telephone interviewer. We recorded and collected the following information: demographics (age and sex), origin of the infection, physiological parameters (vital signs and laboratory data), and clinical outcomes. Acute physiology and chronic health evaluation (APACHE) II and modified sequential organ failure assessment (SOFA) scores were used to evaluate the severity. Platelet count was a routine examination and was obtained by analyzing blood samples at admission. Vital signs and laboratory tests were performed at the time of admission. The sepsis state, APACHE II, and SOFA scores were evaluated by experienced physicians and nurses after admission.

### 2.3. Statistical analysis

SPSS software (version 22.0; SPSS, Inc., Chicago, Illinois) was used to perform all statistical analyses. Data were first assessed for normality. Normally distributed quantitative data are expressed as mean and standard deviation, and non-normally distributed quantitative data are presented as median (interquartile range). Categorical data were shown as numbers and percentages. Student *t* test and Mann–Whitney *U* test were used to determine the difference between the 2 groups of normally and non-normally distributed variables, respectively, as appropriate. For categorical variables, we used the χ^2^ or Fisher exact test appropriately to find the difference between groups. Univariate and subsequent multivariate logistic regression analysis were conducted to evaluate the risk factors for mortality of sepsis. We obtained the corresponding odds ratios (ORs) and 95% confidence interval (CIs) of each factor. Receiver operating characteristic (ROC) curves were used to determine the predictive value of platelet and the constructed model. In addition, we used the Z test to find the difference between these 2 ROC curves. A 2-sided *P* value of < .05 was considered to indicate statistical significance.

## 3. Results

### 3.1. Participant characteristics and intergroup comparisons

As shown in Figures 1, 391 patients with sepsis were admitted to the ICU. In total, 101 patients were excluded because they were aged < 18 years (n = 25), had acute bleeding (n = 18), missing data on hospital mortality and platelet count at admission (n = 15), hematological malignancy (n = 12), or administration of any affecting drugs (n = 31). After exclusion, 290 patients with sepsis were included in the study (Table 1). In the present study, the mortality rate of sepsis was 37.9%. We divided these patients into survivor (n = 180) and nonsurvivor (n = 110) groups. Compared with survivors, nonsurvivors tended to be much older and had significantly higher APACHE II and SOFA scores (*P* < .001). There were no differences in the origin of sepsis and vital signs, including mean arterial pressure, heart rate, and body temperature on admission between the 2 groups. In consideration of laboratory tests, compared with the survivor group, we found that the nonsurvivor group had a lower level of hemoglobin (*P* = .011), Pao2/Fio2 (*P* < .001), whereas it had a higher level of D-dimer (*P* = .004), creatinine (*P* < .001), cystatin c (*P* < .001), and lactate (*P* < .001). The platelet count was significantly lower in the nonsurvivor group than in the survivor group (*P* < .001). There was no significant difference in the length of ICU stay (ICU LOS) between the 2 groups (*P* = .298); however, the survivor group had a longer length of hospital stay (hospital LOS) (*P* < .001).

**Table 1:** Baseline characteristics of the survivors group and nonsurvivors group.

Variables	Total cohort (n = 290)	Survivors (n = 180, 62.1%)	Nonsurvivors (n = 110, 37.9%)	*P* value
Age (yr), mean (SD)	59.2 (17.0)	56.4 (16.8)	64.5 (16.0)	<.001
Gender, male, *n* (%)	185 (63.8%)	121 (67.2%)	64 (58.2%)	.12
APACHE II, mean (SD)	21.2 (7.5)	18.8 (7.2)	25.2 (6.2)	<.001
SOFA, mean (SD)	8.8 (4.4)	7.5 (4.1)	11.2 (3.7)	<.001
Origin of sepsis, *n* (%)				
Pulmonary	187 (71.4%)	113 (62.8%)	74 (67.3%)	.483
Abdominal	76 (26.2%)	50 (27.8%)	26 (23.6%)	.436
Other	27 (9.3%)	17 (9.4%)	10 (9.1%)	.92
Vital signs in admission				
Mean arterial pressure (mm Hg), median (IQR)	71 (65, 93)	71 (65, 98)	69 (64, 82)	.168
Heart rate (bpm), median (IQR)	114 (100, 132)	113 (100, 129)	114 (99, 136)	.652
Body temperature (°C), median (IQR)	37.8 (36.8, 38.5)	37.9 (37.0, 38.5)	37.4 (36.7, 38.5)	.112
WBC (×10^9^/L), median (IQR)	12.9 (8.3, 17.5)	12.9 (8.6, 16.8)	12.9 (8.3, 18.9)	.691
Platelet (×10^9^/L), median (IQR)	61.0 (34.0, 114.0)	94.0 (47.0, 143.0)	41.5 (27.0,61.1)	<.001
D-dimer (mg/L), median (IQR)	6.6 (3.6, 13.0)	5.3 (3.5, 10.8)	7.8 (4.4, 17.5)	.004
Fib (g/L), mean (SD)	3.6 (1.7)	3.7 (1.7)	3.3 (1.6)	.053
Hemoglobin (g/L), mean (SD)	99.6 (25.8)	101.5 (25.5)	93.7 (25.6)	.011
Pao2/Fio2 (mm Hg), median (IQR)	191.1 (124.7, 262.3)	207.3 (153.9, 278.2)	145.4 (86.4212.7)	<.001
Albumin (g/L), mean (SD)	28.6 (5.7)	29.6 (5.3)	27.1 (5.9)	<.001
Creatinine (μmol/L), median (IQR)	85.0 (58.0, 167.5)	73.0 (53.0,118.8)	129.0 (66.0, 238.6)	<.001
Cystatin c (mg/L), median (IQR)	1.17 (0.83, 1.99)	0.97 (0.77,1.40)	1.82 (1.07, 2.78)	<0.001
Cholesterol (mmol/L), median (IQR)	2.4 (1.7, 3.2)	2.4 (1.6, 3.3)	2.4 (1.8, 2.9)	.891
HDH (IU/L), median (IQR)	0.5 (0.3, 0.9)	0.5 (0.3, 0.8)	0.6 (0.3, 0.9)	.319
LDH (IU/L), median (IQR)	1.0 (0.5, 1.6)	0.9 (0.5, 1.7)	1.0 (0.5, 1.5)	.963
Lactate (mmol/L), median (IQR)	2.2 (1.4, 3.5)	1.75 (1.30, 3.20)	2.8 (1.6, 4.9)	<.001
Chlorine (mmol/L), mean (SD)	105.6 (8.51)	105.6 (7.55)	105.5 (9.9)	.886
Glucose (mmol/L), median (IQR)	8.1 (6.4, 10.7)	8.1 (6.4, 10.3)	8.7 (6.4, 11.5)	.429
PT (s), median (IQR)	14.6 (13.1, 16.9)	14.5 (13.1, 16.6)	15.1 (13.1, 19.1)	.143
ICU LOS (d), median (IQR)	13 (6, 23)	12 (6, 21)	15 (6, 24)	.298
Hospital LOS (d), median (IQR)	25 (15, 42)	30 (18, 47)	17 (7, 31)	<.001

Pao2/Fio2, ratio of partial pressure of arterial oxygen (Pao2) to inspired fraction of oxygen (Fio2).

APACHE = acute physiology and chronic health evaluation, HDH = high density lipoprotein, ICU = intensive care unit, LOS = length of stay, LDH = lactic dehydrogenase, PT = prothrombin time, SD = standard deviation, SOFA = sequential organ failure assessment, WBC = white blood cell.

### 3.2. Risk factors of mortality in patients with sepsis

Multivariate logistic regression analysis was used to determine independent risk factors for mortality. To identify the risk factors of mortality in patients with sepsis, we included statistically significant variables in the baseline comparison and possible risk factors of outcomes. After adjustment for age, APACHE II, SOFA, platelet, D-dimer, hemoglobin, Pao2/Fio2, albumin, creatinine and lactate levels, the independent risk factors for mortality in patients with sepsis were an increased age (OR = 1.037, 95% CI: 1.012–1.016, *P* = .003), decreased platelet count (OR = 0.983, 95% CI: 0.975–0.991, *P* < .001) and increased lactate levels (OR = 1.217, 95% CI: 1.035–1.431, *P* = .018) (Table 2).

**Table 2:** Univariate and multivariate logistic regression analysis of risk factors associated with mortality in patients with sepsis.

Variabales	Unadjusted analysis			Adjusted analysis		
	OR	95% CI	*P* value	OR	95% CI	*P* value
Age	1.027	1.012–1.042	.001	1.037	1.012–1.061	.003
Gender, male	0.678	0.415–1.108	.121			
APACHE II, mean (SD)	1.142	1.097–1.189	<.001	1.050	0.978–1.127	.179
SOFA, mean (SD)	1.233	1.154–1.318	<.001	1.085	0.950–1.239	.229
Mean blood pressure	0.992	0.981–1.003	.165			
Heart rate	1.003	0.997–1.008	.337			
Body temperature	0.838	0.679–1.035	.102			
WBC	1.014	0.988–1.041	.287			
Platelet	0.981	0.974–0.987	<.001	0.983	0.975–0.991	<.001
D-dimer	1.026	1.003–1.049	.025	1.021	0.991–1.052	.175
Hemoglobin	0.998	0.978–0.997	.012	0.988	0.974–1.002	.101
Pao2/Fio2	0.995	0.992–0.998	.001	0.999	0.995–1.002	.450
Albumin	0.924	0.884–0.966	<.001	0.945	0.889–1.004	.068
Creatinine	1.002	1.001–1.004	.005	0.999	0.996–1.001	.238
PT	1.013	0.981–1.045	.437			
Lactate	1.269	1.125–1.432	<.001	1.217	1.035–1.431	.018

Pao2/Fio2, ratio of partial pressure of arterial oxygen (Pao2) to inspired fraction of oxygen (Fio2).

APACHE = acute physiology and chronic health evaluation, CI = confidence interval, HDH = high density lipoprotein, ORs = odds ratios, PT = prothrombin time, SD = standard deviation, SOFA = sequential organ failure assessment, WBC = white blood cell.

### 3.3. Predictive value of platelet count and constructed model on mortality in patients with sepsis

**Figure 2.**
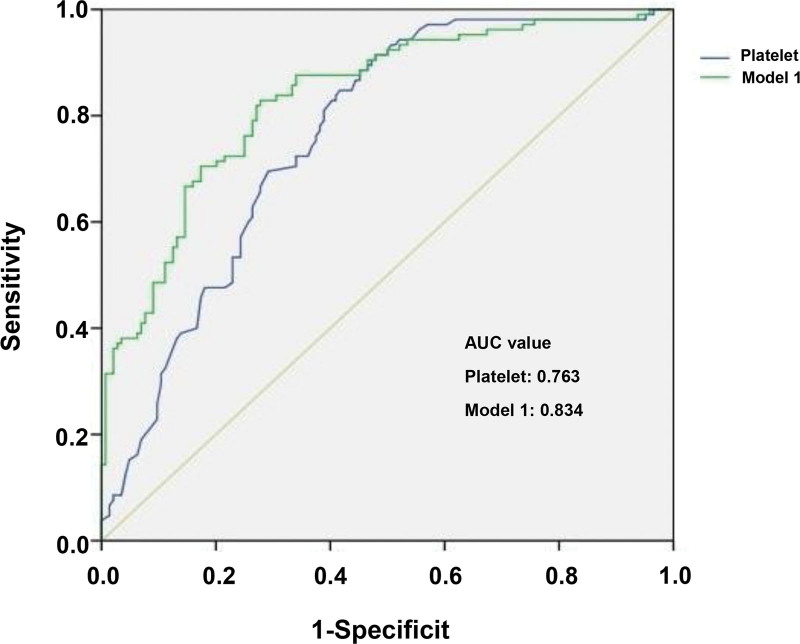
ROC curve analysis comparing the predictive value of platelet and model 1 for mortality in patients with sepsis. Model 1 was composed of age, platelet and lactate. The AUC values of platelet and model 1 were 0.763 (95% CI, 0.709–0.817, *P* < .001) and 0.834 (95% CI, 0.783–0.884, *P* < .001), respectively. There was no significant difference between platelet and model 1 (Z = 0.252, *P* > .05). AUC = area under the receiver operating characteristics curve, CI = confidence interval, ROC = receiver operating characteristics curve.

We used 3 statistically significant factors, namely age, platelet count and lactate level, to construct the predictive model (model 1) for mortality in patients with sepsis. The odds ratios and 95% confidence intervals for each factor were showed in Table 3. In addition, The ROC curves of platelets and model 1 (predictive model composed of age, platelets and lactate) were drawn, and their respective values in predicting mortality in patients with sepsis were compared (Table 4). The results demonstrated that the cut off level of platelet count was 84 and the area under the ROC curve of platelet was 0.763 (95% CI, 0.709–0.817, *P* < .001), with a sensitivity of 55.6% and a specificity of 91.8% for the prediction of mortality in patients with sepsis. The area under the ROC curve of Model 1 was 0.834 (95% CI, 0.783–0.884, *P* < .001) (Table 4). There was no significant difference between the platelet count and model 1 (Z = 0.252, *P* > .05) (Fig. 2).

**Table 3:** Multifactor model 1 to predict mortality in patients with sepsis shock.

Variables	OR	95% CI	*P* value
Age	1.041	1.021–1.061	<.001
Platelet	0.980	0.972–0.988	<.001
Lactate	1.301	1.133–1.493	<.001

The model consists of 3 factors that are statistically significant in multivariate logistic regression analysis.

CI = confidence interval, OR = Odds ratio.

**Table 4:** Comparison of AUC value between platelet and Model 1.

Variables	AUC	95% CI	Sensitivity	Specificity	Cutoff value	*P* value
Platelet	0.763	0.709–0.817	0.556	0.918	84	<.001
Model 1	0.834	0.783–0.884	0.829	0.722		<.001

AUC = area under the receiver operating characteristics curve, CI = confidence interval.

## 4. Discussion

Our study, which included 290 consecutive patients with sepsis, suggested that the platelet count at admission between survivor and nonsurvivors with sepsis varied greatly, and a drop in platelet count at admission was associated with higher mortality. Age, platelet count and lactate level have been shown to be independent risk factors for mortality in patients with sepsis.

TP was found commonly in patients with sepsis during the course of ICU stay.^[14,15]^ In sepsis, platelets become activated, play crucial roles in immunity, and modulate physiological responses to inflammation and infection.^[16,17]^ They interact with innate immune cells and exert immunomodulatory effects via the release of chemokines and cytokines.^[18]^ Involved in both inflammation and thrombosis, platelets contribute to an excessive inflammatory host response and promote the development and progression of sepsis. The mechanisms of TP in the ICU are numerous, and multifactorial mechanisms often act simultaneously, mainly because of a lack of production and excessive consumption, including abnormal megakaryocyte counts caused by bone marrow suppression, disseminated intravascular coagulation, immune-mediated destruction of platelets, and direct and indirect platelet activation during sepsis.^[17,19,20]^ However, the presence of an increase in thrombopoietin (TPO) levels in septic patients with low platelet counts^[21]^ suggests that the rate of platelet consumption is faster than that of sepsis.

The critical role of platelet count in sepsis has been highlighted by its inclusion as a key component in the SOFA score and other scores associated with sepsis severity.^[22]^ TP is associated with an increased risk of sepsis severity and poor prognosis, which may be explained by the contribution of platelets to the pathophysiology of sepsis.^[23,24]^ It has been reported that severe TP is independently associated with disease severity and mortality at the ICU admission and is associated with a dysregulated host response.^[25]^ However, some studies indicate that a single platelet count measurement is not predictive of mortality.^[14,26]^ The discrepancy in these reported values may be associated with patient heterogeneity, different stages of infection, inclusion criteria, host response, pathogens, and other factors. Indeed, platelet count changes in sepsis may have a biphasic pattern, usually characterized by an initial drop followed by an increase and then thrombocytosis.^[14]^ However, some severe sepsis patients may lack this biphasic response and present with persistent TP, which is associated with poor prognosis and increased mortality.^[27]^ In the present study, a drop in platelet count at admission was associated with higher mortality in patients with sepsis, possibly because the patients enrolled in this study had severe TP and were in the late-stage of infection. Nijsten et al^[28]^ reported that a blunted or absent reversal in platelet count in critically ill patients was associated with increased mortality.

Our study had several limitations. First, this was an observational study conducted at a single center. The subjects included were mainly late-stage septic patients with moderate-to-severe TP who were treated in the ICU. Therefore, it is difficult to avoid selection bias. Second, the study was retrospective and we recorded only the platelet count at admission but not fluctuations of platelet count, which only allowed us to show statistical associations and not causality between the change in platelet count and hospital mortality. Third, although multivariable logistic regression was used to adjust for potential confounders, many other potential confounding factors might also exist, leading to biased results. Despite these limitations, the finding that the platelet count is valuable and convenient for predicting mortality is relatively credible.

## 5. Conclusion

In this retrospective observational study, we found that age, platelet count and lactate level were independent risk factors for mortality in patients with sepsis, and a decrease in platelet count at admission was associated with higher mortality. Although the model consisting of age, platelet count and lactate level is a better indicator for predicting mortality in patients with sepsis, platelet count as a single biomarker also showed good predictability in determining the prognosis of patients with sepsis, which can be easily measured and applied. Larger, high-quality randomized controlled trials are needed to reevaluate these findings.

## Author contributions

**Conceptualization:** Yusi Hua, Ruoran Wang, Jie Yang, Xiaofeng Ou.

**Formal analysis:** Yusi Hua, Ruoran Wang, Xiaofeng Ou.

**Methodology:** Yusi Hua, Ruoran Wang, Jie Yang, Xiaofeng Ou.

**Supervision:** Xiaofeng Ou.

**Visualization:** Yusi Hua, Xiaofeng Ou.

**Writing – original draft:** Yusi Hua.

**Writing – review & editing:** Yusi Hua, Ruoran Wang, Jie Yang, Xiaofeng Ou.
